# The 100 most impactful articles on the rotator cuff: an altmetric analysis of online media

**DOI:** 10.1186/s40634-022-00530-7

**Published:** 2022-09-12

**Authors:** Brett D. Haislup, William R. Rate, Matthew D. Civilette, Andrew S. Cohen, Blake M. Bodendorfer, Heath P. Gould

**Affiliations:** 1 MedStar Union Memorial Hospital, Baltimore, MD USA; 2 Georgetown University School of Medicine, Washington, DC, USA; 3 University of Chicago, Chicago, IL USA; 4 Miller Orthopedic Specialists, Council Bluffs, IA and Omaha, NE, USA

## Abstract

**Purpose:**

The purpose of our study was to use the Altmetric Attention Score (AAS) to evaluate the 100 most impactful articles in online media pertaining to the rotator cuff and compare their characteristics to the most-cited rotator cuff articles in the scientific literature.

**Methods:**

We performed an article extraction using Altmetric Explorer to identify all published articles pertaining to the rotator cuff. The top 100 articles with the highest AAS were included for analysis. Several data elements were extracted for each included article: title, article type, article topic, year of publication, journal name, authors, institutional affiliations, and online mentions (i.e. the number of times the article was mentioned in news, blog, Twitter, Facebook, and Wikipedia sources). The geographic origin of each article was also determined by the institutional affiliation of the first author, which was categorized as American (originating in the United States), European (originating in Europe), or other.

**Results:**

The 100 articles with the highest AAS were published between 2009 and 2020, with AAS ranging from 47 to 676 (median: 74.5, 25^th^ percentile: 59.5, 75^th^ percentile: 114.5). Of all online media sources, Twitter correlated most strongly with AAS (*r* = 0.9007, *r*^*2*^ = 0.8112). The selected articles were most frequently published in the American Journal of Sports Medicine (13), the Journal of Shoulder and Elbow Surgery (11), and the Journal of Bone and Joint Surgery (7). The most common article type was Systematic Review/Meta-Analysis (29%), followed by Randomized Controlled Trial (15%). The top 3 AAS articles were all published by authors based in Europe.

**Conclusion:**

The most impactful rotator cuff articles in online media generated substantial online attention. These studies were often performed in Europe and tended to be high level of evidence, focusing on treatment of rotator cuff pathology. The rotator cuff articles that produced the most online attention differed from a previous report of the most-cited rotator cuff articles, suggesting that alternative metrics may be used in concert with conventional bibliometrics to obtain a more complete representation of scientific impact.

## Introduction

Rotator cuff disorders — ranging across the spectrum from rotator cuff tendonitis and subacromial bursitis to degenerative and traumatic rotator cuff tears — are common musculoskeletal problems that affect 30–50% of people over 50 years of age [1]. Given the strong prevalence of rotator cuff dysfunction in the general population, it follows that the rotator cuff has been one of the most researched topics in orthopaedic shoulder surgery to date [2]. Previous studies have reported the most-cited articles pertaining to the rotator cuff [3–5]. However, citation scoring and other conventional bibliometric indicators do not account for the engagement, such as article viewership that is accessed through social media posting and online media production, or advertisements on YouTube. Critical analysis of literature is important as it helps authors to recognize the amount of viewership and who is accessing their articles. Ultimately, researchers are assessed by the quality and quantity of their work in academia, to help guide promotion to tenured positions, which was traditionally evaluated in part by journal impact factor [6]. There are new or alternative metrics that are now able to assess these alternative modes of engagement.

The Altmetric Attention Score (AAS) provides a quantitative assessment of the impact of scholarly articles online and in various forms of social media. The field of orthopaedic surgery has moved toward the utilization of social media as a means of distributing knowledge and exchanging ideas pertaining to musculoskeletal research [7–12]. Previous studies have reported the most impactful articles in online media pertaining to other sports medicine topics such as the anterior cruciate ligament [13]. However, the authors are not aware of any prior study that has used AAS to evaluate the rotator cuff articles that generate the greatest online attention.

The purpose of our study was to evaluate the 100 most engaged articles in online media pertaining to the rotator cuff and compare their characteristics to the most-cited rotator cuff articles in the scientific literature, using the Altmetric Attention Score. Our goal was to achieve a better understanding of the online dissemination of the rotator cuff research as an added dimension of rotator cuff research impact. Secondarily, we compared the predominant features of these articles to previous reports of the most-cited rotator cuff articles in the literature [4, 5]. Our overarching goal was to achieve a more comprehensive understanding of the impact of rotator cuff research that extends beyond the realm of conventional bibliometric analysis. We hypothesized that the 100 most impactful articles in online media pertaining to AAS would not be influenced by bibliometric data.

## Materials and methods

The Altmetric database was queried on July 20, 2020, to identify articles pertaining to the rotator cuff. The search was performed using the PubMed MeSH terms “rotator cuff” or “supraspinatus” or “infraspinatus” or subscapularis or “teres major” or “teres minor” and yielded 6,183 articles published between 1931 and 2020. These articles were stratified by highest to lowest AAS and the 100 articles with the highest scores were included for analysis. The Altmetric Attention Score is a weighted, automated algorithm that takes into account the quantity of a post’s output and the quality of the post’s source, such as newspaper articles, Twitter posts, blogs, policy documents [14]. These scores can be accessed via the Altmetric Database. All articles that focused on other orthopaedic topics were sequentially excluded until 100 articles were gathered that were directly pertinent to the rotator cuff.

Collected data included title, authors, year of publication, journal name, institutional affiliations, article type, article topic, and online mentions (for example, the number of times the article was mentioned in news, blogs, Twitter, Facebook, and Wikipedia). Article type was identified from the article abstract and classified as original research (subclassified as randomized controlled trial, prospective cohort, retrospective cohort, case–control, case series, case report, or laboratory study), descriptive epidemiology, systematic review/meta-analysis, review, editorial/expert opinion, clinical commentary, or other. Article topics were anatomy, basic science, biomechanics, cost, diagnostics, treatment, epidemiology/risk factors, injury prevention, rehabilitation/return to play, patient satisfaction/quality of life, and other. The geographic origin of the paper was determined by the institutional affiliation of the first author, categorized as American (originating in the United States), European (originating in Europe), or other. The number of citations were provided by Google Scholar (Alphabet, Mountain View, CA), which lists the number of citations for each article.

STATA 15.1 (STATACorp) was used for calculations and statistical analysis. Median and quartiles were calculated for AAS. Spearman correlation and logarithmic regression were used to determine the relationship between online mentions and AAS, and analysis of variance (ANOVA) was used to determine whether article type, topic, or geographic origin were associated with AAS.

## Results

**Fig. 1:**
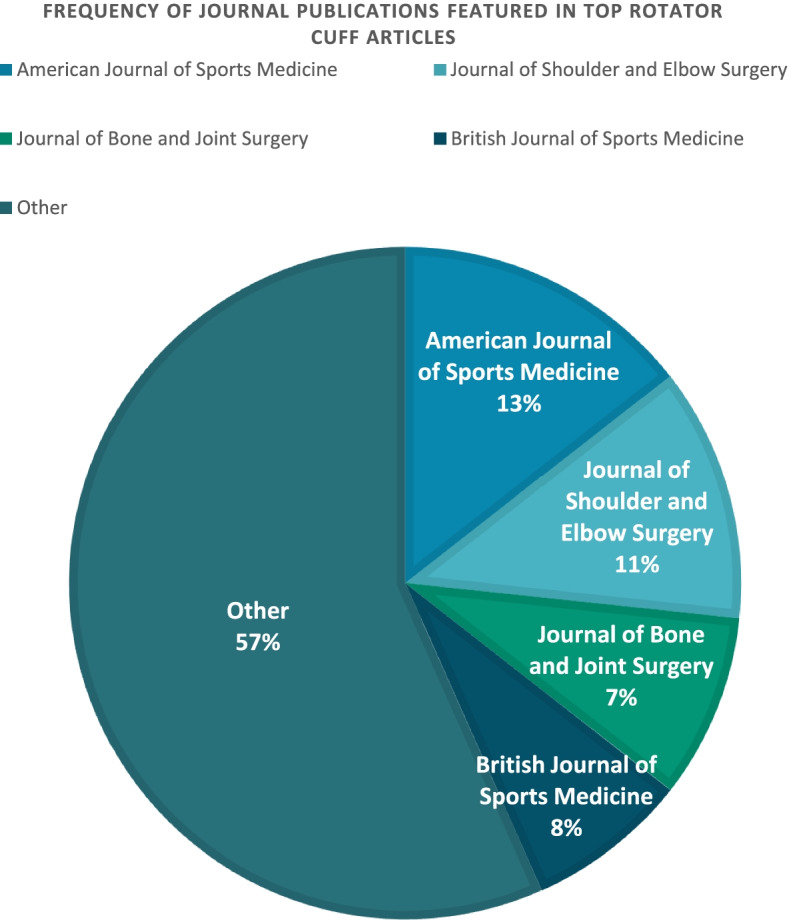
Most frequently published journals for the 100 articles with the top altmetric attention score when searching for “rotator cuff”

The 100 articles with the highest AAS were published between 2009 and 2020, with AAS ranging from 47 to 676 (median: 74.5, 25^th^ percentile: 59.5, 75^th^ percentile: 114.5) (Table 1). The selected articles were most frequently published in the American Journal of Sports Medicine (13), the Journal of Shoulder and Elbow Surgery (11), and the Journal of Bone and Joint Surgery (8) (Fig. 1). The most common article type was Systematic Review/Meta-Analysis (29%), followed by Randomized Control Trial (15%). The most common article topics were Treatment (38%), Epidemiology (19%), and Patient Satisfaction/Quality of Life (10%). Nearly half of the selected articles (44/100, 44%), including the top 3 AAS articles, were published by authors located in Europe. One-quarter of the selected articles (25/100, 25%) were published by authors in the United States, while the remaining articles (31/100, 31%) were published by other.

**Table 1:** Top 25 rotator cuff articles ranked by altmetric attention score

Rank	Altmetric Attention Score	Article Citation	Year Published	Journal	Level of Evidence	Number of Citations
1	676	Lewis J. Rotator cuff related shoulder pain: Assessment, management and uncertainties. Man Ther. 2016 Jun;23:57–68	2016	Manual Therapy	Level VII	109
2	540	Ketola S et al. Arthroscopic decompression not recommended in the treatment of rotator cuff tendinopathy: a final review of a randomised controlled trial at a minimum follow-up of ten years. Bone Joint J. 2017 Jun;99-B(6):799–805	2017	The Bone & Joint Journal	Level I Randomized Control Trial	37
3	441	Nagra NS et al. Mechanical properties of all-suture anchors for rotator cuff repair. Bone Joint Res. 2017 Feb;6(2):82–89	2017	Bone and Joint Research	Level VII	28
4	378	Boorman RS et al. What happens to patients when we do not repair their cuff tears? Five-year rotator cuff quality-of-life index outcomes following nonoperative treatment of patients with full-thickness rotator cuff tears. J Shoulder Elbow Surg. 2018 Mar;27(3):444–448	2018	Journal of Shoulder and Elbow Surgery	Level II Prospective Cohort	24
5	334	Salamh P et al. It Is Time to Put Special Tests for Rotator Cuff-Related Shoulder Pain out to Pasture. J Orthop Sports Phys Ther. 2020 May;50(5):222–225.Epub 2020 Apr 9	2020	Journal of Orthopaedic & Sports Physical Therapy	Level VII	0
6	330	Mohamadi A et al. Corticosteroid Injections Give Small and Transient Pain Relief in Rotator Cuff Tendinosis: A Meta-analysis. Clin Orthop Relat Res. 2017 Jan;475(1):232–243. Epub 2016 Jul 28	2016	Clinical Orthopaedics & Related Research	Level I Meta-Analysis	56
7	319	Tamborrini G et al. The Rotator Interval—A Link Between Anatomy and Ultrasound. Ultrasound Int Open. 2017 Jun;3(3):E107-E116. Epub 2017 Aug 23	2017	Ultrasound International Open	Level VII	8
8	246	Lewis J, McCreesh et al. Tendinopathy: Navigating the Diagnosis-Management Conundrum. J Orthop Sports Phys Ther. 2015 Nov;45(11):923–37. Epub 2015 Sep 21	2015	Journal of Orthopaedic & Sports Physical Therapy	Level VII	10
9	238	Applegate KA et al. Association Between Cardiovascular Disease Risk Factors and Rotator Cuff Tendinopathy: A Cross-Sectional Study. J Occup Environ Med. 2017 Feb;59(2):154–160	2017	Journal of Occupational & Environmental Medicine	Level III	11
10	233	McCreesh KM et al. Increased supraspinatus tendon thickness following fatigue loading in rotator cuff tendinopathy: potential implications for exercise therapy. BMJ Open Sport Exerc Med. 2017 Dec 26;3(1):e000279	2017	BMJ Open Sport & Exercise Medicine	Level I	10
11	233	Cook T et al. Are corticosteroid injections more beneficial than anaesthetic injections alone in the management of rotator cuff-related shoulder pain? A systematic review. Br J Sports Med. 2018 Apr;52(8):497–504.Epub 2018 Jan 5	2018	British Journal of Sports Medicine	Level I Systematic Review	31
12	213	Kukkonen J et al. Treatment of non-traumatic rotator cuff tears: A randomised controlled trial with one-year clinical results. Bone Joint J. 2014 Jan;96-B(1):75–81	2014	The Bone & Joint Journal	Level I Randomize d Control Trial	130
13	175	Carr A et al. Effectiveness of open and arthroscopic rotator cuff repair (UKUFF): a randomised controlled trial. Bone Joint J. 2017 Jan;99-B(1):107–115	2017	The Bone & Joint Journal	Level I Randomized Control Trial	54
14	168	Hermans J et al. Does this patient with shoulder pain have rotator cuff disease?: The Rational Clinical Examination systematic review. JAMA. 2013 Aug 28;310(8):837–47	2013	JAMA: Journal of the American Medical Association	Level I Systematic Review	160
15	165	Gumina S et al. Rotator cuff degeneration of the healthy shoulder in patients with unilateral arm amputation is not worsened by overuse. Knee Surg Sports Traumatol Arthrosc. 2018 Jan;26(1):182–187.2. Epub 2017 Jul 13	2017	Knee Surgery, Sports Traumatology, Arthroscopy	Level of Evidence III Cadaveric, Retrospective	2
16	161	Piper CC et al. Operative versus nonoperative treatment for the management of full-thickness rotator cuff tears: a systematic review and meta-analysis. J Shoulder Elbow Surg. 2018 Mar;27(3):572–576. Epub 2017 Nov 21	2018	Journal of Shoulder and Elbow Surgery	Level I	0
17	158	Park JH et al. Effect of Smoking on Healing Failure After Rotator Cuff Repair. Am J Sports Med. 2018 Oct;46(12):2960–2968. Epub 2018 Aug 21	2018	American Journal of Sports Medicine	Level III Retrospective Cohort	17
18	147	Peach MS et al. Engineered stem cell niche matrices for rotator cuff tendon regenerative engineering. PLoS One. 2017 Apr 3;12(4):e0174789	2017	PLoS ONE	Not Applicable	39
19	132	Dunn WR et al. Symptoms of pain do not correlate with rotator cuff tear severity: a cross-sectional study of 393 patients with a symptomatic atraumatic full-thickness rotator cuff tear. J Bone Joint Surg Am. 2014 May 21;96(10):793–800	2014	Journal of Bone & Joint Surgery, American Volume	Level III Retrospective Cohort	119
20	128	Chang KV et al. Early Versus Delayed Passive Range of Motion Exercise for Arthroscopic Rotator Cuff Repair: A Meta-analysis of Randomized Controlled Trials. Am J Sports Med. 2015 May;43(5):1265–73. Epub 2014 Aug 20. Erratum in: Am J Sports Med. 2015 Aug;43(8):NP26	2014	American Journal of Sports Medicine	Level I Systematic Review	68
21	127	Dean BJ et al. Glucocorticoids induce specific ion-channel-mediated toxicity in human rotator cuff tendon: a mechanism underpinning the ultimately deleterious effect of steroid injection in tendinopathy? Br J Sports Med. 2014 Dec;48(22):1620–6. Epub 2014 Mar 27	2014	British Journal of Sports Medicine	Not Applicable	64
22	127	Rawat P et al. Effect of rotator cuff strengthening as an adjunct to standard care in subjects with adhesive capsulitis: A randomized controlled trial. J Hand Ther. 2017 Jul-Sep;30(3):235–241.e8. Epub 2016 Nov 21	2017	Journal of Hand Therapy	Level I Randomized Control Trial	9
23	126	Yamamoto A et al. The impact of faulty posture on rotator cuff tears with and without symptoms. J Shoulder Elbow Surg. 2015 Mar;24(3):446–52. Epub 2014 Oct 16	2015	Journal of Shoulder and Elbow Surgery	Level III Cross Sectional	40
24	123	Kong BY et al. Structural Evolution of Nonoperatively Treated High-Grade Partial-Thickness Tears of the Supraspinatus Tendon. Am J Sports Med. 2018 Jan;46(1):79–86.. Epub 2017 Sep 26	2017	American Journal of Sports Medicine	Level IV Case Series	9
25	115	Ranebo MC et al. Surgery and physiotherapy were both successful in the treatment of small, acute, traumatic rotator cuff tears: a prospective randomized trial. J Shoulder Elbow Surg. 2020 Mar;29(3):459–470. Epub 2020 Jan 7	2020	Journal of Shoulder and Elbow Surgery	Level I Randomized Control Trial	1

Among all 100 articles, there were a total of 16,581 Twitter mentions (mean: 165.81), 1077 Facebook mentions (mean: 10.77), and 212 mentions in mainstream online news outlets (mean: 2.12). The online media source that correlated most strongly with AAS was Twitter mentions (*r* = 0.9007, *r*^*2*^ 2 = 0.8112), whereas the other online media sources demonstrated weak correlations with AAS.

## Discussion

This study identified the 100 most mentioned articles in online media pertaining to the rotator cuff. The characteristics of these impactful articles in online media were correlated with Twitter and Facebook citations and had a high proportion of European authorship, with publication in frequently cited American journals. AAS provides a unique dimension of article impact that may be used as an adjunct to citation analysis.

The top 100 rotator cuff articles in online media were published in a wide range of journals. Although the *American Journal of Sports Medicine*, *Journal of Shoulder and Elbow Surgery*, and *Journal of Bone and Joint Surgery* were the most common, these three journals only combined for about one-third of all articles in the top 100. *AJSM* was also the most represented journal among the most impactful articles in online media pertaining to the ACL, but the *AJSM*-published articles accounted for a much larger proportion of articles in those studies (44% and 34%, respectively) than in the present study examining the rotator cuff (13%) [7]. These findings suggest that a larger number of journals seem to be actively disseminating rotator cuff research in online media, compared to other major research topics in the field of sports medicine. One contributing factor to this trend may be the fact that ACL injuries tend to affect competitive athletes, whereas rotator cuff dysfunction often affects the general non-athlete population [15, 16]. This could conceivably lead to a lower proportion of the most impactful articles on the rotator cuff being published in subspecialty-specific journals that are focused on sports medicine. This trend parallels the findings of prior studies that have reported a high proportion of the most cited rotator cuff articles published in the *Journal of Bone and Joint Surgery*, [4, 17] a journal that includes all topics related to orthopaedic surgery and is not restrictive to sports medicine or shoulder surgery. Further investigation is needed to improve our collective knowledge of how rotator cuff research can be distributed most effectively, both in the arena of scientific publication as well as in online media.

The most common article types among the top 100 AAS rotator cuff articles were Systematic Review/Meta-Analysis (29%) and Randomized Control Trial (15%), indicating that higher level-of-evidence studies tend to generate the most attention in online media. These findings are consistent with prior studies that have examined the top AAS articles for other sports medicine topics such as the ACL [13]. In contrast, previous studies investigating the most-cited articles on the rotator cuff found that Case Series were the most common article type, [3, 4] likely due to the fact that many of the cited articles are comprised of the early, seminal work on rotator cuff disease, which laid the foundation for the higher level-of-evidence studies that have been conducted more recently and appear to be generating more online attention. This disparity between the most-cited rotator cuff articles and the highest AAS rotator cuff articles with regard to article type and level of evidence underscores the utility of alternative metrics in providing a more current, real-time snapshot of the impact of rotator cuff research, as compared to the long-term assessment of impact that can be gleaned from conventional bibliometrics.

The most common article topics in our study were Treatment (38%), Epidemiology (19%), and Patient Satisfaction/Quality of Life (10%). Studies that focus on treatment and epidemiology are helpful to healthcare providers because they enhance the understanding of which patients are most likely to get injured and how to provide optimal care for them. Patient satisfaction was the third most common topic in regard to rotator cuff, whereas rehabilitation and return to play were more common in ACL reconstruction. These disparate results are likely due to the fact that ACL injuries are more relevant to athletic individuals, whereas rotator cuff injuries commonly occur in non-athletes who would be more concerned with returning to normal life activities rather than returning to sport.

The largest proportion of top 100 AAS rotator cuff articles were published by authors based in Europe (44%), whereas US studies comprised only one-quarter (25%) of the included articles. This finding conflicts with previous studies that have reported a strong predominance of US articles among the most impactful studies in online media pertaining to the ACL (54%) [13]. European authors are widely regarded to be at the forefront of shoulder research, with several of the major developments in the field of shoulder surgery occurring in France (e.g. Goutallier classification, Grammont prosthesis, Latarjet procedure) [18]. Therefore, it is not surprising that much of the online attention surrounding rotator cuff research is derived from European articles. However, it is interesting that our findings regarding geographic origin contrast with previous studies that have reported a majority of US articles (58% and 60%) [4, 19] among the most-cited rotator cuff studies in the literature. The reasons for this disparity are not certain, but it is possible that European authors, journals, and healthcare organizations may be disseminating rotator cuff research more actively in online media compared to their US counterparts. Further investigation is needed to verify this hypothesis, as well as to elucidate any other factors that may be responsible for this finding.

Authors can find their article’s AAS by clicking the Altmetric link on the cite that hosts their publication, which offers a list of platforms that have interacted with their article. The website includes a comparison of engagement with other articles that have been published within the same scientific genre, over a similar timeframe. There is no definitive numerical score that is used as a benchmark to determine if a score is good or bad, since many factors go into calculating these scores. For instance, if a publication is produced this year that publication will be compared to other publications that are produced this year rather than those produced 10 years ago, which would have had greater opportunity to be disseminated through media and a greater opportunity to collect citations [11].

There are several limitations inherent to Altmetric that may have affected the findings of the present study. Altmetric is updated on a daily basis in a semi-continuous fashion; thus, it is possible to identify a different list of top 100 articles depending upon the specific date on which the search was performed. Moreover, Altmetric does not account for context and is solely dependent upon the volume of online mentions. Thus, both negative and positive discussion equally impact AAS, and a high AAS may not necessarily reflect a high quality study [20–23].

## Conclusion

The most impactful rotator cuff articles in online media generated substantial online attention. These studies were often performed in Europe and tended to be high level of evidence, focusing on treatment of rotator cuff pathology. The rotator cuff articles that produced the most online attention differed from a previous report of the most-cited rotator cuff articles, suggesting that alternative metrics may be used in concert with conventional bibliometrics to obtain a more complete representation of scientific impact. Future use of the Altmetric Attention Score may improve authors’ understanding of their readership demographics by considering online dissemination and the timing of article publication, which are unique to AAS.

## References

[1] da Rocha Motta G, Amaral MV, Rezende E, Pitta R, dos Santos Vieira TC, Duarte MEL, Vieira AR, Casado PL (2014). Evidence of genetic variations associated with rotator cuff disease. J shoulder Elb Surg.

[2] Namdari S, Baldwin K, Kovatch K, Huffman GR, Glaser D (2012). Fifty most cited articles in orthopedic shoulder surgery. J shoulder Elb Surg.

[3] Familiari F, Castricini R, Galasso O, Gasparini G, Iannò B, Ranuccio F (2021). The 50 highest cited papers on rotator cuff tear. Arthroscopy.

[4] Kraeutler MJ, Freedman KB, MacLeod RA, Schrock JB, Tjoumakaris FP, McCarty EC (2016). The 50 most cited articles in rotator cuff repair research. Orthopedics.

[5] Lei L, Zhang C, Sun F-H, Xie Y, Liang B, Wang L, Pang G, Chen R, Jiang W, Ou X, Miyamoto A, Wang J (2021). Research trends on the rotator cuff tendon: a bibliometric analysis of the past 2 decades. Orthop J Sport Med.

[6] McKiernan EC, Schimanski LA, Muñoz Nieves C, Matthias L, Niles MT, Alperin JP (2019). Use of the Journal Impact Factor in academic review, promotion, and tenure evaluations. Elife.

[7] Chien JL, Sabharwal J, Namoglu EC, Ghassibi MP, Yuan M, Gandy C, Wei C, Somohano K, Engelhard SB, Petrakos P, Van Tassel SH, Chien G-F, Belyea DA (2021). The 100 most mentioned glaucoma articles online with highest altmetric attention scores. J Glaucoma.

[8] Earp BE, Kuo K, Shoji MK, Mora AN, Benavent KA, Blazar PE (2020). Evaluating the online presence of orthopaedic surgeons. J Am Acad Orthop Surg.

[9] Franko OI (2011). Twitter as a communication tool for orthopedic surgery. Orthopedics.

[10] Hughes H, Hughes A, Murphy C (2017). The use of twitter by the trauma and orthopaedic surgery journals: twitter activity, impact factor, and alternative metrics. Cureus.

[11] Jildeh TR, Okoroha KR, Guthrie ST, Parsons TW (2019). Social media use for orthopaedic surgeons. JBJS Rev.

[12] McLawhorn AS, De Martino I, Fehring KA, Sculco PK (2016). Social media and your practice: navigating the surgeon-patient relationship. Curr Rev Musculoskelet Med.

[13] Civilette MD, Rate WR, Haislup BD, Cohen AS, Camire L, Bodendorfer BM, Gould HP (2022) The top 100 most impactful articles on the anterior cruciate ligament: an altmetric analysis of online media. SAGE Open Med 10:20503121221111694. 10.1177/20503121221111694 10.1177/20503121221111694 PMC9340895 35924141

[14] How is the Altmetric Attention Score Calculated. (2021). Altmetric. https://help.altmetric.com/support/solutions/articles/6000233311-how-is-the-altmetric-attention-score-calculated-

[15] Sanders TL, MaraditKremers H, Bryan AJ, Larson DR, Dahm DL, Levy BA, Stuart MJ, Krych AJ (2016). Incidence of anterior cruciate ligament tears and reconstruction. Am J Sports Med.

[16] Thelwall M, Haustein S, Larivière V, Sugimoto CR (2013). Do altmetrics work? Twitter and ten other social web services. PLoS One.

[17] Familiari F, Castricini R, Galasso O, Gasparini G, Iannò B, Ranuccio F (2021) The fifty highest cited papers in rotator cuff tear. Arthrosc J Arthrosc Relat Surg 37(1):61–68. 10.1016/j.arthro.2020.07.044 10.1016/j.arthro.2020.07.044 32798669

[18] Patil V, Kosmidis I, Tsekes D (2019). A bibliometric analysis: 200 most cited papers in the field of shoulder surgery. JSES Open Access.

[19] Sochacki KR, Jack RA, Nauert R, Harris JD (2018). Correlation between quality of evidence and number of citations in top 50 cited articles in rotator cuff repair surgery. Orthop J Sport Med.

[20] Kelly JC, Glynn RW, O’Briain DE, Felle P, McCabe JP (2010). The 100 classic papers of orthopaedic surgery: a bibliometric analysis. J Bone Joint Surg Br.

[21] Kunze KN, Polce EM, Vadhera A, Williams BT, Nwachukwu BU, Nho SJ, Chahla J (2020). What Is the Predictive Ability and Academic Impact of the Altmetrics Score and Social Media Attention?. Am J Sports Med.

[22] Kwok R (2013). Research impact: Altmetrics make their mark. Nature.

[23] Luc JGY, Archer MA, Arora RC, Bender EM, Blitz A, Cooke DT, Hlci TN, Kidane B, Ouzounian M, Varghese TK, Antonoff MB (2020). Social media improves cardiothoracic surgery literature dissemination: results of a randomized trial. Ann Thorac Surg.

